# 'Pedometers cost buttons': the feasibility of implementing a pedometer based walking programme within the community

**DOI:** 10.1186/1471-2458-11-200

**Published:** 2011-03-31

**Authors:** Rebecca Shaw, Elisabeth Fenwick, Graham Baker, Chloe McAdam, Claire Fitzsimons, Nanette Mutrie

**Affiliations:** 1 School of Medicine, University of Glasgow, 1 Lilybank Gardens, Glasgow G12 8RZ, UK; 2 Health Economics and Health Technology Assessment, Centre for Population & Health Sciences, University of Glasgow, 1 Lilybank Gardens, Glasgow G12 8RZ, UK; 3 School of Psychological Sciences and Health, University of Strathclyde, Jordanhill Campus, 76 Southbrae Drive, Glasgow G13 1PP, UK

## Abstract

**Background:**

Recent studies have suggested that walking interventions may be effective (at least in the short term) at increasing physical activity amongst those people who are the most inactive. This is a leading objective of contemporary public health policy in the UK and worldwide. However, before committing money from limited budgets to implement walking interventions more widely in the community, policymakers will want to know whether similar impacts can be expected and whether any changes will be required to the process to ensure uptake and success. This paper utilises the findings from a recent community-based pedometer study (Walking for Wellbeing in the West - WWW) undertaken in Glasgow, Scotland to address issues of feasibility.

**Methods:**

An economic analysis of the WWW study assessed the costs of the interventions (minimal and maximal) and combined these with the effects to present incremental cost-effectiveness ratios (cost/person achieving the target of an additional 15,000 steps/week). A qualitative evaluation, involving focus group discussions with WWW participants and short interviews with members of the WWW research team, explored perceived benefits and barriers associated with walking, as well as the successful aspects and challenges associated with the interventions.

**Results:**

The incremental cost effectiveness associated with the interventions was estimated as £92 and £591 per person achieving the target for the minimal and maximal interventions respectively. The qualitative evaluation gave insight into the process by which the results were achieved, and identified several barriers and facilitators that would need to be addressed before implementing the interventions in the wider community, in order to ensure their effective transfer. These included assessing the impact of the relationship between researchers and participants on the results, and the motivational importance of monitoring and assessing performance.

**Conclusions:**

The results suggest that pedometer based walking interventions may be considered cost-effective and suitable for implementation within the wider community. However, several research gaps remain, including the importance and impact of the researcher/participant relationship, the impact of assessment on motivation and effectiveness, and the longer term impact on physical and mental health, resource utilisation and quality of life.

**Trial registration:**

Current Control Trials Ltd ISRCTN88907382

## Background

Promoting physical activity, particularly among the most inactive, is a leading aim of contemporary public health policy in the UK [1,2]. Walking has been identified as the mode of physical activity most likely to appeal to inactive people [2,3]. A recent systematic review of interventions to promote walking concluded that interventions targeted at the individual, such as brief advice, telecommunications and supported use of pedometers "could contribute substantially towards increasing the activity levels of the most sedentary"[4]. There is a growing body of evidence to suggest that pedometers, combined with a goal-setting programme, can be an effective short term motivational tool to increase walking by between 2,000 and 2,500 steps/day [5,6]. However, the systematic review of walking interventions [4] also noted that most existing research provides evidence of short term efficacy (less than 12 weeks) rather than longer term effectiveness, and there remain few community-based studies of pedometer interventions that provide long-term follow-up greater than 12 months [5]. In addition, studies are often based on small, convenience or volunteer samples and so provide little evidence about what happens once the programme has been adopted and implemented more widely [4].

Policymakers seeking to spend money from limited budgets to improve physical activity amongst the most inactive, will be curious to know whether they can expect walking interventions to be effective should they be adopted more widely in the community. In addition, they will be keen to ascertain the most appropriate form of these interventions to implement and whether there are any changes to the intervention and/or implementation process that could impact positively on the uptake and success of the intervention once it is promoted more widely in the community.

The aim of this paper is to utilise information generated from a recent community-based pedometer study (Walking for Wellbeing in the West - WWW) undertaken in Glasgow, Scotland. The study involved four core elements (behavioural, psychological, health and environmental evaluations) and two supplementary studies (economic and qualitative evaluations). This paper focuses on the findings of the supplementary studies, to address these issues of concern to the policymaker and to identify any gaps in the evidence base. In this way, the paper corresponds to recent Medical Research Council (MRC) guidance for evaluating complex interventions which suggests the importance of evaluating both outcomes and process in order to address "a key question in evaluating complex interventions [of]... whether they are effective in everyday practice" [7].

In what follows, we introduce the community-based study and detail the methods employed for the supplementary economic and qualitative evaluations. The paper summarises the results of these studies and discusses the potential impact of these results for policymakers considering the widespread introduction of a pedometer based walking programme.

### Community-based pedometer study

Walking for Wellbeing in the West (WWW) found that an individualised, pedometer based intervention was effective in initiating (at 12 weeks) and maintaining (at 12 months) walking behaviour change in Scottish men and women who were not achieving the recommendations for physical activity at baseline [8-10]. The study involved seventy-nine participants (women = 63, age = 49.2 ± 8.9 years) randomly assigned between two walking interventions - maximal (n = 39, women = 31) and minimal (n = 40, women = 32). For an initial period of 12 weeks, the minimal intervention group constituted a waiting list control and were requested to maintain normal levels of walking.

The maximal intervention involved the participants receiving an individualised walking programme over 12 weeks, a pedometer (Omron HJ-109e) and an individual physical activity consultation (average 30 minutes) with a trained member of the research team. The consultation was based upon the transtheoretical model of exercise behaviour change [11] and was structured to enhance motivation and help participants develop strategies to increase their walking (for example, producing walking plans and goals) and to overcome barriers (for example, identifying social support). At the end of the 12 week intervention, participants receiving the maximal intervention had a follow-up, individual physical activity consultation (average 30 minutes), also based on the transtheoretical model [11]. This second consultation focused on strategies to avoid relapse and maintain activity levels. Further material provided to the maximal intervention group included a physical activity advice leaflet (at 24 weeks) and a follow-up consultation (average eight minutes) via the telephone (at 36 weeks). The aim of this additional support was to remind participants of the benefits of maintaining their walking behaviour.

After 12 weeks, the waiting list control group received the minimal intervention. This involved participants receiving an identical, individualised walking programme (also over 12 weeks) and a pedometer, but no 30 minute individual physical activity consultation. Instead, the minimal intervention involved brief advice concerning goal setting and self monitoring (average five minutes) at the start of the intervention. At the end of their 12 week structured programme (week 24) and 12 weeks later (week 36) the minimal intervention group each received a short (average five minutes) individual feedback session relating to their current levels of walking and use of the pedometer (rather than the 30 minute follow-up physical activity consultation received by the maximal intervention group). Participants in the minimal intervention group did not receive any of the additional material described above. For further details on the study rationale and design see Fitzsimons et al. (2008) [10].

Analysis of post intervention data (12 weeks) for the maximal intervention group showed increased walking and improved mood (Positive and Negative Affect Schedule), both statistically significant compared with baseline and the waiting list control group [8]. Follow-up at 12 months for both maximal and minimal intervention groups suggests that improvements over baseline values in walking and positive affect were achieved and sustained in both groups, despite a reduction in step counts from levels achieved immediately post intervention. There was no statistically significant difference between groups [9].

## Methods

### Economic evaluation

Economic evaluation compares the additional cost associated with a new programme/policy (for example, the introduction of a pedometer based walking intervention) with the additional outcomes achieved as a result of the change (for example the change in weekly step count), compared to the alternative programme(s) available. The aim is to inform resource allocation decisions by assessing the value for money of the programme/policy change and determining whether the change is a good use of scarce resources compared to the alternative ways of using those resources.

The economic analysis of WWW sought to assess the costs and outcomes, measured over 12 months, associated with the maximal and minimal interventions, as well as with "usual behaviour" as represented by the waiting list control. The aim of the analysis was to determine whether a pedometer based walking intervention could be considered cost-effective and, if so, in which format (minimal or maximal).

The costing was restricted to the resources associated with providing the intervention. Table 1 details the resource use and unit costs associated with the maximal and minimal interventions. The assumption was made that there are no costs associated with the "usual behaviour" group. Unit cost values were taken from published estimates [12] or estimated from the costs incurred by the trial centre.

**Table 1:** resource use and unit costs

	Resource use		Cost
	
	Minimal intervention	Maximal Intervention	Unit cost per unit or hour
Pedometer	1	1	£ 13.00

Individual physical activity consultation (mins)	5	30	£ 17.50

Walking programme	1	1	£ 1.00

Individual relapse prevention consultation (mins)	5	30	£ 17.50

Physical activity advice leaflet		1	£ 0.16

Follow-up call (mins)	5	8	£ 17.50

The measure of outcome employed in the economic analysis for both the maximal and minimal interventions is the number of participants achieving and maintaining a target of an additional 15,000 steps/week from pre-intervention levels (i.e. baseline for maximal intervention and week 12 for minimal intervention). This target equates to the public health guidance of achieving 150 minutes of moderate-intensity activity over the course of a week [13], under the assumption that moderate intensity walking is achieved by a step rate of approximately 100 steps/min [14,15]. The effect of "usual behaviour" is determined from the waiting list control as the number of participants achieving a target of an additional 15,000 steps/week between baseline and week 12 under the assumption that if these participants had continued to receive no intervention they would maintain that level of walking.

Cost-effectiveness results are typically presented as incremental cost-effectiveness ratios (ICERs) which identify the additional costs associated with an intervention per additional unit of outcome generated by the intervention, compared to standard treatment (see equation 1 below).(1)

For WWW, the ICER is presented in terms of the additional costs associated with the provision of the intervention per additional person achieving the target of a weekly increase of ≥ 15,000 steps. As the chosen measure of outcome is specific to walking interventions, it does not enable comparison of cost-effectiveness to interventions in other areas (for example, screening or smoking cessation). In order to allow such comparisons an outcome measure common to all interventions is required. Quality Adjusted Life Years (QALYs) are frequently used in economic analyses of healthcare interventions to provide a common measure of outcome. QALYs present the impacts of interventions on both quality and quantity of life in a single, common metric; thus enabling comparison between interventions in different areas of health care. As such, in addition to presenting the cost-effectiveness in terms of cost per target achiever, a sensitivity analysis was undertaken to determine the amount of QALYs that the interventions would need to generate in order for them to be considered cost-effective at the usual cost-effectiveness thresholds specified for the UK (£20,000 - £30,000/QALY [16]). For example, if an intervention led to one additional person achieving the target at a cost of £10,000, then a person achieving the target would have to generate at least 0.5 QALYs to be considered cost-effective at £20,000/QALY or ⅓ QALYs to be considered cost-effective at £30,000/QALY.

### Qualitative study

The qualitative evaluation involved four focus group discussions with participants, and short interviews with six members of the research team. The focus groups were convened in order to gain an in-depth understanding of participants' views and experiences of the study and a "valuable insight....into why a successful intervention works and how it can be optimised"[17]. Typically, a focus group involves a small number of people (four to eight) in an informal group discussion focused around a particular set of issues. The discussion is usually based on a series of questions (the focus group schedule) and the researcher generally acts as a moderator for the group [18].

In this study, separate focus groups were conducted with the minimal and maximal intervention groups at the end of the intervention period (i.e. 12 weeks for maximal and 24 weeks for minimal) and again at 12 month follow-up. The focus group schedule explored the perceived benefits of increased walking; views on the pedometer, the physical activity consultations and on-going support (for maximal intervention); barriers encountered; future recommendations and reflections on participation in the study. There was also an opportunity (at the end of the discussion) for feedback on topics of importance to participants that were not covered elsewhere. Participants were recruited via information sheets posted to their home addresses. All focus groups took place in a private room at the study centre and were facilitated by the lead author (who is an experienced facilitator). Anonymity and confidentially were assured. Each group was attended by three to six participants, lasted for approximately an hour and was audio-recorded (with permission). The groups consisted of more women than men, and generally involved more individuals who had met their targets than those who had not (referred to as 'high' and 'low' adherers below). See Table 2 for more details on the participant breakdown.

**Table 2:** characteristics of focus groups

	Gender	Average age	Adherence
Focus group 1 - Maximal intervention (12 weeks)	4 women, 1 man	54 (min 45; max 60)	5 High

Focus group 2 - Minimal intervention (24 weeks)	4 women, 2 men	54 (min 37; max 63)	5 High and 1 Low

Focus group 3 - Maximal intervention (12 months)	2 women, 1 man	53 (min 38; max 63)	1 High and 2 Low

Focus group 4 - Minimal intervention (12 months)	3 women	55 (min 51; max 61)	3 High

The short, semi-structured interviews with members of the research team (undertaken by the lead author), covered the aspects of WWW that the researchers thought had been successful, the challenges of WWW and how feasible the researchers thought it would be to implement something similar across the wider community. Each of the six key members of the research team who were interviewed had been involved in implementing and coordinating the intervention.

Focus groups and interviews were transcribed verbatim (and anonymised) by the lead author and thematically analysed. The process of thematic analysis involves "identifying, analysing and reporting patterns (themes) within data"[19]. Computer software (ATLAS.ti) was used in order to facilitate the analysis. First, initial codes were identified, based on careful reading and re-reading of the data. These codes were then sorted into potential themes. Direct quotes from the data were grouped under thematic headings [20], providing a clear illustration of each theme and also some indication of the frequency with which each theme was addressed. Finally, the themes were refined through repeated investigation both of similar and anomalous examples [21]. Quotations were chosen to illustrate particular points and are identified in the text below by an anonymised code (indicating focus group (FG) or researcher interview (R), gender and whether 'high' or 'low' adherer).

### Ethical approval

Appropriate ethical approval was attained from the University of Strathclyde ethics committee and all procedures were carried out in accordance with the Declaration of Helsinki.

## Results

### Economic evaluation

Table 3 presents the results of the economic analysis, detailing the costs, outcomes and cost-effectiveness (in terms of cost per number of participants achieving the target of a weekly step increase of ≥15,000 steps) associated with "usual behaviour", minimal and maximal intervention.

**Table 3:** Cost-effectiveness results

	Total cost of intervention per group	Number achieving weekly target ≥15,000 steps	Incremental cost	Incremental outcome	Incremental cost/target achiever
"Usual behaviour"	£ -	4	£ -	-	-

Minimal intervention	£ 735	12	£ 735	8	£ 92

Maximal intervention	£ 1,326	13	£ 591	1	£591

The results suggest that maintaining "usual behaviour" (i.e. no intervention), as assessed by the waiting list control over 12 weeks, leads to four participants achieving a weekly step increase of ≥15,000 steps at, an assumed, zero cost. Minimal intervention results in an additional eight participants achieving the target at an extra cost of £735. Thus, the cost-effectiveness of the minimal intervention compared to "usual behaviour" is an additional £92 per additional target achiever (= 735/8). Maximal intervention results in 13 participants achieving the target (one more than with the minimal intervention) at a cost of £1,326 (£591 more than the minimal intervention). Thus, the ICER associated with the maximal intervention, compared to the minimal intervention, is £591 per additional target achiever (£591/1).

These results suggest that either intervention (minimal or maximal) may be considered cost-effective. The decision between which of the two to adopt would depend on the societal value placed on each person achieving the target. For example, when the value of a person achieving the target is rated between £92 and £590, then the minimal intervention would be considered cost-effective. In contrast, if the value placed on a person achieving the target was £591 or more, then the maximal intervention would be considered cost-effective. However, if the value placed on a person achieving the target was less than £92 neither intervention would be considered cost-effective.

In the UK, standard thresholds used to determine cost-effectiveness are based on a societal value for a QALY (λ). These values are typically in the region of £20,000-£30,000/QALY [16]. A sensitivity analysis was carried out to assess how many QALYs would need to result from each person achieving the target for each of the interventions to be considered cost-effective according to these standards (i.e. the weight that would need to be attached to each target achiever for the ICER of the intervention to fall below these standard values). This is determined for each intervention by dividing the ICER associated with that intervention by the standard level of cost-effectiveness required (λ).(2)

Thus, in order for the minimal intervention to be considered cost-effective at a level of £30,000/QALY, achieving and maintaining the target of ≥ 15,000 additional steps per week over 12 months would need to improve each person's lifetime QALYs by at least 0.0031 (= 92/30000). If achieving and maintaining the target over 12 months increased each person's lifetime QALYs by more than 0.02 (= 591/30000), then the maximal intervention would be considered cost-effective against this standard threshold. This level of increase in QALYs equates to an increase in survival (in full health), as a result of the maximal intervention, of 7.3 days over a lifetime (= 0.02*365), or 1.1 day over a lifetime for the minimal intervention (= 0.0031*365).

### Qualitative evaluation

The data from the four focus groups with participants (FG) and the six semi-structured interviews with research staff (R) were combined and analysed together, and are presented below in terms of three themes representing: 1) support, 2) monitoring and 3) practical issues.

#### Support

Throughout the project, two researchers were primarily responsible for delivering the intervention and co-ordinating assessments. Each researcher took responsibility for half of the participants and maintained regular contact for up to 12-15 months:

*We got to know them, they got to know us. We asked about their family etcetera *(R1)

Participants were very positive about the support they received from these two researchers. So much so, that as the quote below shows, they were anxious about what they would do when the support stopped:

*If I'm not in the project, I think I'll lose motivation completely. I don't think I'll be able to continue walking if I'm not in the project *(FG 2, male, high adherer)

Indeed, although the quantitative outcomes showed that participants maintained increased walking after 12 months in comparison to baseline [8,22], in focus group discussions a number of participants spoke of a lack of motivation, with some also reporting the strategies they had used to overcome this:

*Once I'd handed in the book and didn't have the book anymore, that sort of took away some of the pressure and my walking dropped off *(FG 2, female, high adherer)

*I think writing down what you've done is really useful. Since we've stopped I've been writing it down in a chart I've drawn myself *(FG 3, female, high adherer)

#### Monitoring

The majority of respondents felt that the step-count provided by the pedometer provided useful feedback which supported and encouraged them:

*I think the pedometer was really useful at the beginning because you got tuned into it and checked it and I realised that my days at home and days at work were really quite different so I needed to make a special effort on my days off *(FG 4, female, high adherer)

When initially designing the study, concerns were expressed by the research staff about the level of participant burden, particularly in terms of measurement of health outcomes such as body mass and cholesterol (which necessitate physical measurements and blood being taken). In fact, for those who participated in the study, these health checks were considered an incentive for their initial and continued involvement in the study:

*I wanted to do it because I could get my weight monitored and so on; so I could see if it was actually having any impact *(FG 4, female, high adherer)

*I really would have liked to have results every time we met. That's the main reason I took part ((blood pressure etc.)) *(FG 4, female, high adherer)

#### Practical issues

Many of the participants said that walking appealed over other forms of activity because it was cost-free, could be undertaken alone without generating feelings of self-consciousness and could fit easily into their daily routine:

*I like [walking] because I can do it by myself and I don't have to embarrass myself at the gym. I can afford to do it because it doesn't cost anything and I can do it when I'm going to the shops or work *(FG 2, female, high adherer)

In the first pair of focus groups (taking place shortly following the 12 week intervention), all but one of the participants had achieved their targets and they had done this either by making time for walks during the day ("I try to take a walk every day" (FG 1, male, high adherer)), or by incorporating it into their daily activities:

*I haven't set out to walk everyday; instead I've tried to incorporate it into what I usually do. So rather than taking the bus to ((Place)), I'll now walk and it means taking much more time to actually get there *(FG 1, female, high adherer)

Participants also mentioned the physical, social and emotional benefits of increasing walking:

*Yes, when I drop off my daughter and go for a walk I feel invigorated. It makes you wonder why you don't do it more *(FG 1, female, high adherer)

*On my way home I park up at the Botanic Gardens and go for a walk. And I've seen things I didn't know about. I didn't know there was a disused railway track in the Botanic Gardens! It's a great way of getting out there and seeing things that I hadn't done before and meeting the gardeners you know, when you stop for a breath *(FG 1, female, high adherer)

Respondents mentioned a number of barriers to increased walking, such as bad weather ("I hit a rough patch when we had that wet period", FG 1, male, high adherer) and boredom, especially amongst those who had tried to initiate a walk during the day, outside their usual daily activities. In addition, those who'd tried to incorporate walking into ordinary daily activities, like walking to the shops or to work, mentioned barriers such as lack of time and practical issues such as carrying shopping, laptops etc.:

*I found it quite hard walking to work at times. When I have meetings or whatever and I have a laptop to carry, I'm wearing a suit and heels, I just can't do it. So I jump in the car. I'm still trying to take the stairs at work, but more often than not I drive to work now. I mean, it sounds very vain, but I straighten my hair every morning and if it's bad weather, or even a bit damp and I walk, my hair is a frizzy mess by the time I get to work *(FG 3, female, low adherer)

## Discussion

The results from the economic analysis suggest that pedometer based walking interventions, as trialled in the WWW study, may be considered cost-effective depending on the monetary value placed on, or the QALY weight attached to, a person achieving the target. The sensitivity analysis suggests that if the minimal intervention could generate just one additional day of full health it would be considered cost-effective by established standards in the UK. The 12 month effectiveness data indicates that while walking levels fell from those seen immediately post-intervention, increased walking over baseline values can be maintained over a longer time period. If this continued to be the case then impacts on physical health, such as blood pressure, inflammatory markers or weight which were not observed in this group over the short term [8,23], may start to materialise either due to improvements or due to reductions in the extent of decline in these levels over time.

The results from the qualitative evaluation provide information for policymakers about the process by which the study results were achieved and give insight into the barriers and facilitators that need to be acknowledged or addressed before implementing the intervention in the wider community. Some of the issues (for example, the impact of the relationship between researchers and participants on the results) will be specific to the study, but others (for example, the trade-off between walking and commuting) may apply to other walking or physical activity projects more generally. These issues need to be considered in order to determine whether the results from the study are likely to be replicated in the wider community.

When asked whether they thought it would be feasible to implement something similar in the community, one researcher said:

*Super feasible, I don't see any problem at all. Pedometers cost buttons; I think it would be really easy to run, especially without having people to come in for assessments *(R3)

Although the interventions are straightforward and the underlying concept is simple (a pedometer based walking programme and relatively brief advice), a major part of the intervention's success may be due to the support and expertise provided by the researchers working on the WWW study. As shown in the qualitative analysis above, the sustained contact between participant and researcher, necessary for maintaining follow-up in the study setting, may itself have provided motivation to participants and was certainly appreciated by them. In addition, the qualitative analysis showed that the health checks were also perceived to be a motivating factor.

It is not clear how much of the effectiveness of the interventions should be attributed to these contacts and assessments, or whether the removal of such contacts and assessments would have a critical impact on the effectiveness of the interventions. In this case, while the intervention would be "really easy to run... without having people to come in for assessments" (R3), it may not provide the same outcomes. Further research is needed to explore delivery of on-going individual level support, as provided by the two researchers in this study, on a wider scale. In addition, it should be noted that the economic evaluation of WWW did not take full account of this level of input from the researchers and did not include the costs of assessment (which were seen to be driven by the study protocol). Thus, in order to replicate this level of cost-effectiveness in the community, the implementation would require a similar level of effectiveness from the interventions without the contact and assessments (i.e. there would need to be no impact of the contact and assessments on the effectiveness).

In the post intervention focus groups, some participants suggested ways in which this support could be provided differently, in particular highlighting walking groups. The Scottish Physical Activity Research Collaboration (SPARColl, http://www.sparcoll.org.uk) is currently evaluating the feasibility of wider implementation of the WWW interventions with support provided via community and workplace health walks [24]. This work will include process and outcome evaluation methods which may help to ascertain the level of support required by participants and walk leaders, to successfully implement the intervention in the community.

### Limitations with this analysis

In the initial analysis plan, as described in Fitzsimons et al. (2008) [10], the economic evaluation was due to include the differences in costs resulting from changes in NHS resource use between the interventions and the waiting list control ("usual behaviour"). Unfortunately, it was not possible to estimate the impact of the intervention on healthcare resource use (for example, hospitalisations and GP consultations) as this data was only collected for the maximal and minimal groups post intervention. As such, the slight reduction in resource use for the maximal intervention group identified at 12 months, compared to both the "usual behaviour" control and minimal intervention groups, may simply reflect differences present at baseline, rather than changes in the use of these resources that related to the intervention. The exclusion of these costs limits the analysis to focus only on the short term costs of providing the intervention and potentially underestimates the cost-effectiveness of maximal intervention compared with both minimal intervention and "usual behaviour".

Despite the availability of EQ-5D data, from which estimates of within study quality adjusted life years (QALYs) could be calculated, the primary economic analysis reported here involved the number of participants achieving the target of ≥ 15,000 additional steps per week. This is because an analysis based on the QALYs generated within the trial period (i.e. 12 months), would require the assumption that there was no long term impact of the interventions on either length or quality of life. Given that increased physical activity is associated with reductions in obesity, cardio-vascular disease and cancer [1] this seems an unnecessarily restrictive assumption. Alternatively, an analysis based on lifetime QALYs gained would require the determination of the impacts of the interventions on both length and quality of life. This, in turn, would involve identifying and modelling the complex long term impacts of the interventions on various aspects of health and quality of life, for which there is currently very little data. The reliance on a measure of outcome specific to walking/physical activity interventions limits the ability of the policymaker to compare the cost-effectiveness of the interventions to that of interventions in other areas of health. The sensitivity analysis goes someway to address this by providing an indication of the quality of life impact required for the interventions to be considered cost-effective by the standards usually employed in the UK [16].

## Conclusion

The results of the economic and qualitative analyses of the WWW study suggest that pedometer based walking interventions may be considered cost-effective and suitable for implementation within the wider community. However, several research gaps remain, including the importance and impact of the researcher/participant relationship, the impact of assessment on motivation and effectiveness, and the longer term impact on physical health, resource utilisation and quality of life. Some of these gaps should be addressed by the implementation study currently underway in Glasgow [24], while others will require studies with longer follow-up, such as those recently commissioned by the National Institute for Health Research (NIHR) Health Technology Assessment (HTA) programme.

## Competing interests

The authors declare that they have no competing interests.

## Authors' contributions

RS designed and carried out the qualitative evaluation. EF designed and carried out the economic evaluation. RS and EF drafted the manuscript. GB, CMcA, CF and NM conceived of the WWW study and reviewed and revised the manuscript. All authors read and approved the final manuscript.

## Pre-publication history

The pre-publication history for this paper can be accessed here:

http://www.biomedcentral.com/1471-2458/11/200/prepub
